# Supplementary material for: Characterization of the Menin-MLL Interaction as Therapeutic Cancer Target
Source: Cancers (Basel). 2020 Jan 14;12(1):201. doi: 10.3390/cancers12010201 (PMC7016952; doi:10.3390/cancers12010201)
Supplement: Supplementary file 1 [file cancers-12-00201-s001.pdf]

	Gene Name	Probe Nr.
TaqMan probe	CD11b	Hs00167304_m1
	MNDA	Hs00935905_m1
	18s RNA	4319413E
	MEIS1	Hs00180020_m1
	GREB1	Hs00536409_m1
	EGR3	Hs00231780_m1
	PGR1	Hs00172183_m1)
	KLK2	Hs00428383_m1
	FKBP5	Mm00487406_m1
	TMPRSS2	Hs00237175_m1

Supplementary Table S1. TaqMan probes used for qRT-PCR

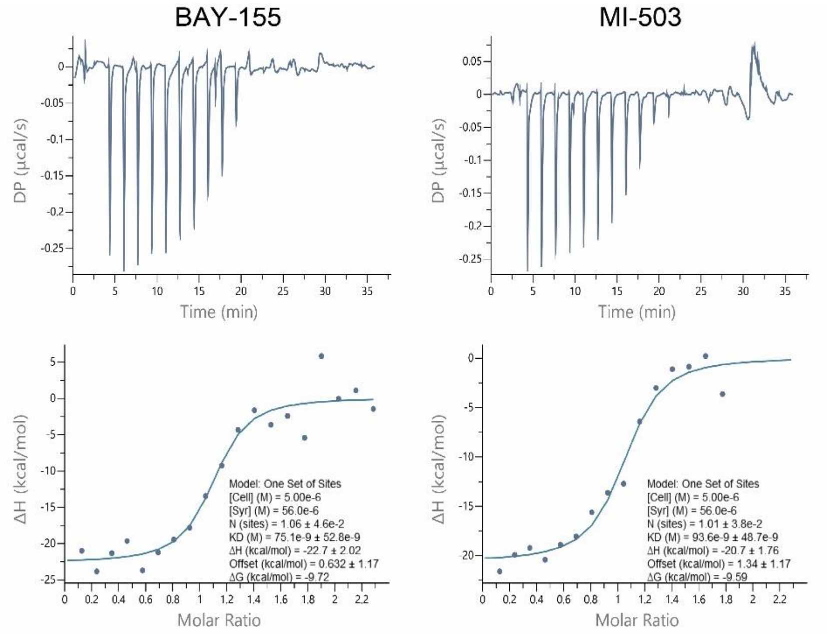

Supplementary Figure S1. Isothermal Titration Calorimetry measurement of BAY-155 and MI-503 on purified menin protein (1-615 aa).

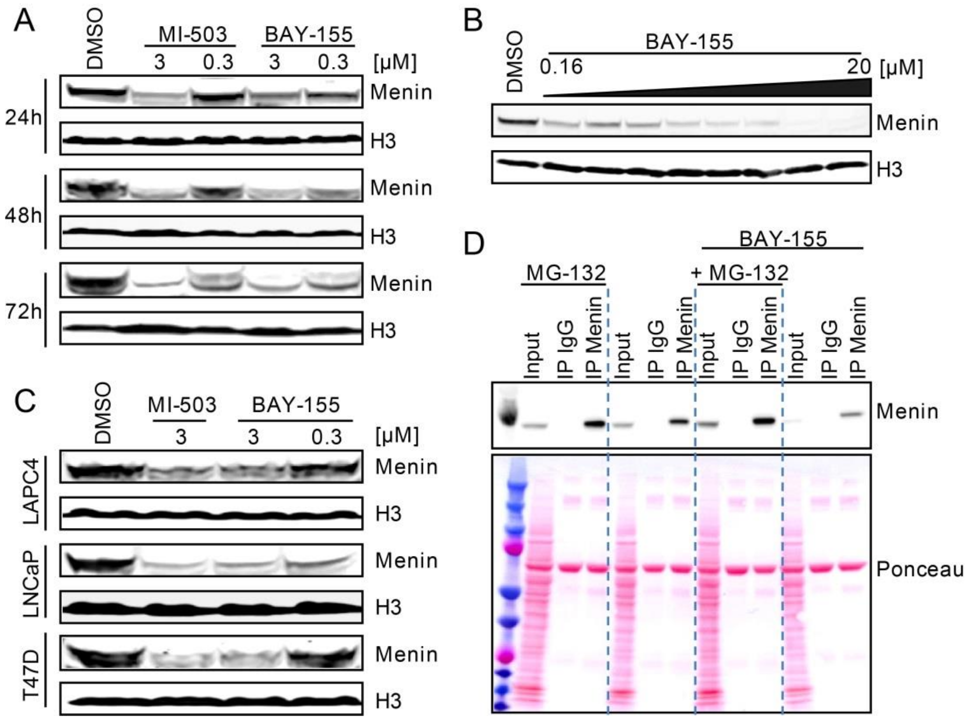

Supplementary Figure S2. BAY-155 treatment leads to degradation of menin protein. (**A**) Western blot depicting menin and histone H3 protein expression after treatment with DMSO, BAY-155 or MI-503 in VCaP cells for the indicated periods of time and concentrations. (**B**) Western blot of menin and histone H3 protein expression in LAPC4, LNCaP and T47D cell lines. (**C**) Western blot showing dose-dependent down-regulation of menin protein expression after BAY-155 treatment in VCaP cells. (**D**) Rescue of menin protein degradation with MG-132 co-treatment. Western blot of menin immunoprecipitation after DMSO, MG-132 (10 μM), BAY-155 (10 μM) or BAY-155 (10 μM) together with MG-132 (10 μM) treatment in VCaP cells for 16 h. Ponceau staining was used as loading control.

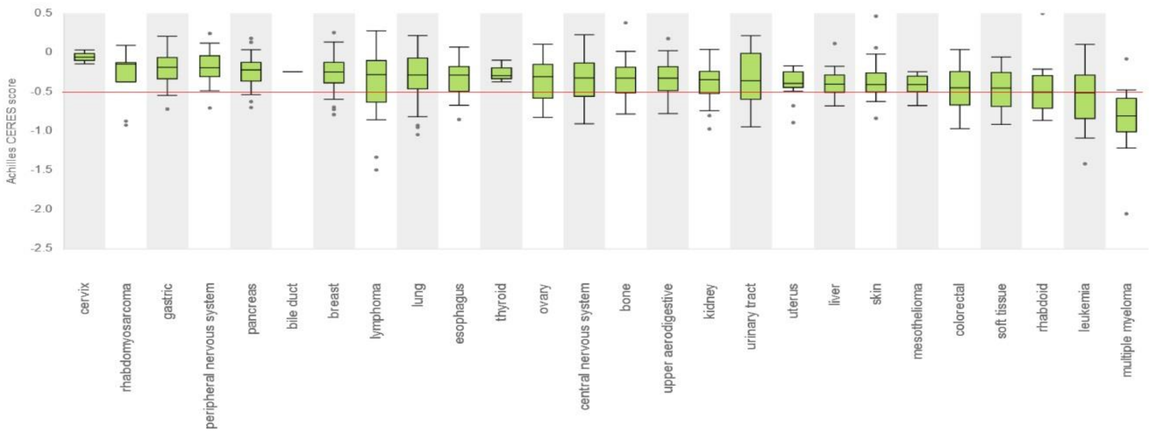

Supplementary Figure S3. Effects of MEN1 knock-out in the Achilles Project data set.

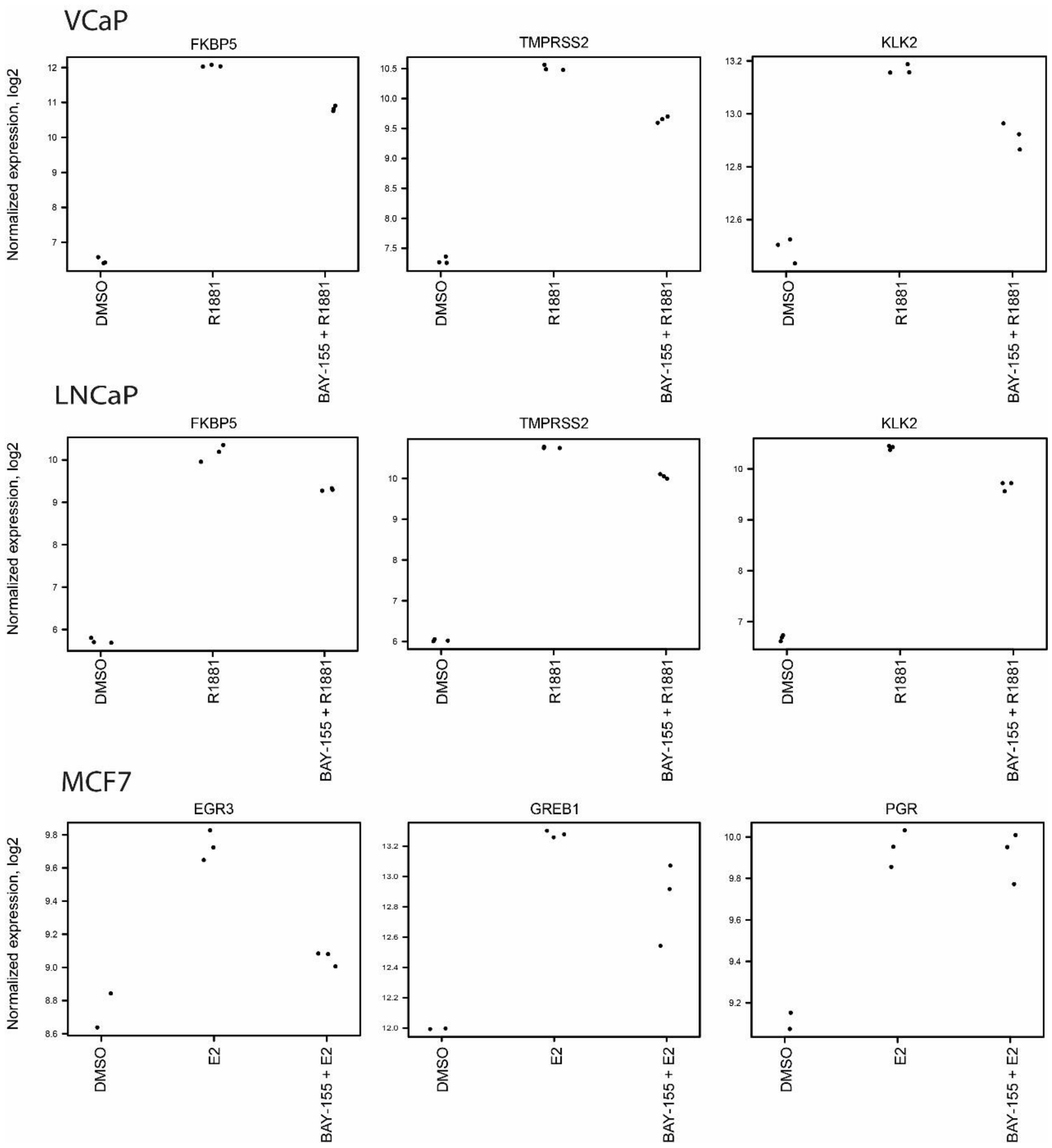

Supplementary Figure S4. Differential gene expression effects of BAY-155 on androgen and estrogen target genes. Data are presented as normalized log2 expression values of biological replicates.
